# Understanding the implications of the Sustainable Development Goals for health policy and systems research: results of a research priority setting exercise

**DOI:** 10.1186/s12992-019-0534-2

**Published:** 2020-01-09

**Authors:** Sara Bennett, Nasreen Jessani, Douglas Glandon, Mary Qiu, Kerry Scott, Ankita Meghani, Fadi El-Jardali, Daniel Maceira, Dena Javadi, Abdul Ghaffar

**Affiliations:** 1 Johns Hopkins Bloomberg School of Public Health, 615 N Wolfe St, Baltimore, MD 21205 USA; 2 Africa Center for Evidence (ACE), University of Johannesburg, Johannesburg, South Africa; 3 Centre for Evidence Based Health Care (CEBHC), Stellenbosch University, Stellenbosch, South Africa; 4 American University of Beirut, Beirut, Lebanon; 5 Center for the Study of State and Society, Buenos Aires, Argentina; 6 Alliance for Health Policy and Systems Research, World Health Organization, Geneva, Switzerland

**Keywords:** Sustainable Development Goals, Health policy and systems research, Priority setting, Multisectoral collaboration, Social protection, Social accountability

## Abstract

**Background:**

Given the paradigmatic shift represented by the Sustainable Development Goals (SDGs) as compared to the Millennium Development Goals - in particular their broad and interconnected nature - a new set of health policy and systems research (HPSR) priorities are needed to inform strategies to address these interconnected goals.

**Objectives:**

To identify high priority HPSR questions linked to the achievement of the Sustainable Development Goals.

**Methods:**

We focused on three themes that we considered to be central to achieving the health related SDGs: (i) Protecting and promoting access to health services through systems of social protection (ii) Strengthening multisectoral collaborations for health and (iii) Developing more participatory and accountable institutions. We conducted 54 semi-structured interviews and two focus group discussions to investigate policy-maker perspectives on evidence needs. We also conducted an overview of literature reviews in each theme. Information from these sub-studies was extracted into a matrix of possible research questions and developed into three domain-specific lists of 30–36 potential priority questions. Topic experts from the global research community then refined and ranked the proposed questions through an online platform. A final webinar on each theme sought feedback on findings.

**Results:**

Policy-makers continue to demand HPSR for many well-established issues such as health financing, human resources for health, and service delivery. In terms of service delivery, policy-makers wanted to know how best to strengthen primary health care and community-based systems. In the themes of social protection and multisectoral collaboration, prioritized questions had a strong emphasis on issues of practical implementation. For participatory and accountable institutions, the two priority questions focused on political factors affecting the adoption of accountability measures, as well as health worker reactions to such measures.

**Conclusions:**

To achieve the SDGs, there is a continuing need for research in some already well established areas of HPSR as well as key areas highlighted by decision-makers. Identifying appropriate conceptual frameworks as well as typologies of examples may be a prerequisite for answering some of the substantive policymaker questions. In addition, implementation research engaging non-traditional stakeholders outside of the health sector will be critical.

## Background

### Evolution from the Millennium Development Goals to the Sustainable Development Goals

The adoption of the Sustainable Development Goals (SDGs) in 2016 by the global community marked a radical shift in direction from the previous Millennium Development Goals (MDGs) [1]. While the MDGs reflected an economical list of relatively narrow targets that addressed low and middle income countries (LMICs) alone, the SDGs are more numerous and reflect a more holistic understanding of the nature of sustainable development and its interactions with human health, environmental protection and social justice [2]. While in many respects the MDGs were highly successful in driving international and national investments, and the world’s focus upon the identified goals, they had also been criticized for being insufficiently inclusive in their process of development (in particular in terms of including representatives from the most affected contexts), providing insufficient justification for why they focused on the issues that they did, and for neglecting environmental issues [3]. The SDGs sought to address these criticisms. They were negotiated in a far more consultative fashion using 13 rounds of discussion within the Open Working Group, they clearly address environmental issues, and they are much broader in terms of the targets identified.

For the MDGs, the World Health Organization (WHO) established an international task force to identify health systems research priorities [4]. The Task Force employed an interpretive approach, relying largely on the views of its members to identify a number of broad priority topics including for example: human resources for health at the district level and below; drugs and diagnostic policies; governance and accountability. Since the work of the Task Force, there have been several further efforts, supported by the Alliance for Health Policy and Systems Research, based at the WHO, to engage policy and decision-makers so as to define global priorities for specific domains of health policy and systems research (HPSR) including health financing [5], human resources for health [6], access to medicines [7] and the private health sector [8]. These exercises have drawn upon policy-maker consultations and overviews of research reviews to identify relevant research questions across the various sources. These research questions have then undergone a priority ranking process by researchers, thus providing considerably greater specificity than the broad domains identified by the Task Force.

### Health policy and systems research and the link to the SDGs

HPSR is an applied field, oriented around solving real-world problems, where the value of research findings is inextricably linked to their relevance to policy and decision-making. Accordingly, research priority setting processes which engage policy- and decision-makers in identifying the key challenges that they face in order to frame and prioritize research questions are helpful in ensuring that scarce research funding is used most efficiently [9].

The wide-ranging and interconnected nature of the SDGs may make it challenging to identify how best to act from any one sectoral (education, agriculture, health, environmental sustainability, etc.) perspective. However, this same quality of the SDGs also presents an opportunity for creative and innovative approaches, including for those working in or with the health sector. Goal 3 on health encompasses a large number of disease or condition-specific areas (maternal and child health, infectious diseases, non-communicable diseases, injuries, substance-abuse, road traffic accidents) as well as cross-cutting or systems-related issues including universal health coverage, health financing, human resources for health and disease surveillance. The combination of targets under Goal 3 mean that a narrow focus upon a handful of specific health conditions and the systems needs related to them is no longer a viable health systems strengthening strategy, instead we need to consider how different approaches to health systems strengthening may intersect with multiple different health conditions. Moreover, the non-health goals have many points of connection with health, for example Goal 1 on Ending Poverty includes targets that concern the development of social protection systems and access to basic services; Goal 10 on inequalities addresses empowerment, including for disabled people; Goals 6 and 11 both address aspects of providing clean water, effective sanitation and waste management. The last two, cross-cutting goals on Peace, Justice and Strong Institutions (Goal 16) and Partnerships for the Goals (Goal 17) have implications for health systems given their focus on, among other things, developing effective, accountable and transparent institutions; ensuring responsive, inclusive, participatory and representative decision-making; ensuring public access to information; and capacity building.

Given the paradigmatic shift represented by the SDGs, a new set of HPSR priorities were needed to inform the unique thinking required to address these interconnected goals.

### The need for HPSR priority setting in the SDG era

While other authors had written about the need for research linked to the SDGs [10–13], there had been no other systematic effort to identify health research priorities linked to the goals. A preliminary literature review through searches in PubMed and Google Scholar that combined terms related to (i) the Sustainable Development Goals (ii) health or health systems and (iii) research, yielded 28 papers. Most (*n* = 14) of the papers were commentaries and editorials expressing the views of the authors and frequently seeking to raise the profile of a particular issue (cardiovascular health, eye health, violence against women, health research in Africa) within the context of the SDGs. A further set of more analytical papers (*n* = 11) either reviewed the literature around particular topics related to the SDGs (such as strategies for scaling up early childhood development, or universal health coverage and the SDGs) or analyzed the SDGs themselves. There were just a handful of empirical papers, often quite loosely linked to the SDGs, even if their titles suggested otherwise. Of the 28 papers identified, Universal Health Coverage (UHC) was a central theme of many [14–20]. Additional papers addressed non-communicable diseases (NCDs) which had been entirely absent in the MDGs [12, 21–24], achieving political priority as a precursor for domestic funding [10, 15, 24, 25] and engagement with private sector actors to address inappropriate marketing of harmful products and/or access to life saving drugs and technologies [24, 26, 27]. Overall, we concluded, that there had been little rigorous research or analysis exploring HPSR needs associated with the SDGs, and further, there was not a coherent approach or shared understanding of the likely implications of the SDGs for HPSR.

Given the breadth and complexity of the SDGs already described, establishing an appropriate starting point for a research priority setting exercise was not straightforward. While many have suggested that UHC is the preeminent target within the health goal [18], prior research priority setting efforts, such as those on health financing [5], have already substantively addressed this topic. The SDGs have also cast a spotlight on previously neglected health priorities including NCDs [24], road traffic injuries [21] and mental health [22], but focusing an HPSR priority setting effort around such health topics felt counterintuitive and likely unable to capture the potential paradigm shift that the SDGs represent for HPSR. That said, the research team seriously considered a focus upon understanding and addressing the health system challenges associated with NCDs, including strategies for improving continuity of care, supporting self-care within the household, and strengthening community care systems. However, we decided instead to try to identify as starting points for the research-priority setting exercise, themes that captured the spirit of the SDGs - in particular their focus upon inclusion and social justice, as well as their integrated and connected nature. Drawing upon existing literature and consultations with key stakeholders, including the Alliance HPSR, we identified three themes that distilled some of the unique and innovative attributes of the SDGs, did not overlap with previous priority setting efforts, and offered new potential directions for HPSR. These three themes are:

#### Theme 1 - protecting and promoting access to health services through systems of social protection

SDG 1 on poverty explicitly identifies the importance of systems of social protection, that is, systems that help prevent and mitigate risks related to unemployment, social exclusion, sickness, disability and old age. SDG 1 also acknowledges the critical role that systems of social protection can play in enhancing access to services. The health systems community has placed a high priority on achieving UHC. While this objective is often viewed within the relatively narrow context of the health sector, in practice, UHC can probably only be achieved in conjunction with broader systems of protection that help protect against the risk of impoverishment and facilitate access to complementary social services. Within this domain, we were interested in exploring what kind of research on systems of social protection could help inform the target of achieving UHC.

#### Theme 2 - strengthening multisectoral collaborations for health

Many of the non-health goals acknowledge health aspects within their targets, reflecting growing appreciation of the importance of factors such as urban environment, early childhood education, patterns of food consumption, and changes in the environment and climate on human health. Multisectoral collaboration involves purposeful engagement with actors beyond the health sector to address complex challenges that may undermine health and well-being. While there is growing recognition of the importance of multisectoral collaboration [28, 29], to date it has not been a substantive area for research within HPSR. We were interested in what kind of research could inform the development and implementation of effective multisectoral collaborations for health.

#### Theme 3 - developing more participatory and accountable institutions for health

This theme builds directly upon SDG 16 (Promote just, peaceful and inclusive societies), but also reflects a growing interest within health systems [30, 31]. The theme encompasses alternative strategies for strengthening civic engagement and local accountability within health systems to promote transparency, inclusiveness and participation. This theme is also linked to efforts to create more people-centered health systems, meaning health systems that respond holistically to people’s and communities’ needs [32].

Alternative themes that were considered included (i) how to establish greater political priority for health-related SDGs including domestic financing for health, as well as relatively newer services such as early childhood development, that has received considerable attention in the literature [10, 15, 33], and (ii) issues around engaging private sector actors to address some of the commercial drivers of ill health [24, 26, 27]. Ultimately, however, the research team concluded that the three themes presented above better captured the breadth of HPSR concerns. The theme-specific findings of this research have already been published elsewhere [34–36] with considerably more detail than this paper provides. This paper seeks to synthesize the findings across all three themes, so as to reflect more broadly on new directions for HPSR in light of the SDGs.

## Methods

Many of the research priority setting approaches commonly used in health, such as the Child Health and Nutrition Research Initiative [37] and the Combined Approach Matrix [38] are rooted in quantitative algorithms that consider the burden of disease and the extent to which different types of research may address or avert the burden of disease. Such approaches are typically unsuitable for HPSR as there is too great an uncertainty about the complex pathways through which systems strengthening might affect health outcomes, and the magnitude of effects from these interventions. We therefore drew upon a predominantly interpretive approach supported by the Alliance previously in its research priority setting work [9]. The approach is grounded in (i) consultations with policy makers (ii) reviews of the existing literature and (iii) ranking processes or workshops.

The study was a multi-stage participatory process that included an overview of literature reviews in each of the three thematic areas, consultations with policy-makers from around the world, extraction and synthesis of research questions from the two previous sources, and a global digital exercise that initially focused on refining the proposed questions and secondly, ranking them. Preliminary results were shared through open webinars.

While a global exercise of this nature was not meant to drive national and sub-national research priorities, it was anticipated that this study would, at the very least, inform research at these levels.

### Overview of literature reviews

For each of the three themes the research team worked with an informationist to develop a search strategy for PubMed that was adapted for Embase, Scopus, PAIS International, Social Science Abstracts, PsycINFO, WHO Global Health Regional Indexes, and Ovid’s Global Health database. These searches sought to identify all existing academic review articles on the three topics. We decided to focus only on reviews – rather than reviewing original research articles -- to rapidly and pragmatically map the existing research landscape. Furthermore, review articles enabled us to hone in on research gaps as articulated by the authors of the review articles, as they had already assessed the scope of primary research and synthesized the state of evidence.

Table 1 shows the number of papers identified in the initial searches. To make the analysis more manageable, the research team decided to focus on reviews alone: both systematic and non-systematic reviews were included. In the case of multisectoral collaboration for health, multiple reviews included discussion of multisectoral collaboration (for example as part of reviews on NCDs, or nutrition), but did not have this as their primary focus. These reviews were excluded.

**Table 1:** Data on overview of reviews conducted

	Social Protection	Multisectoral collaboration	Participatory and Accountable institutions
Dates included in review	January 2000 to March 2017	January 2000 to March 2017	All literature up to 1 December 2016
Number of articles identified (excluding duplicates)	6211	13,245	2139
Number of reviews included	34	38	34

Reviewers extracted meta-data from the article (e.g. authors, date, title) as well as the questions addressed by the review, a description of interventions, findings from the review, country focus, conceptual contributions, and knowledge gaps or research questions. The data extracted varied slightly depending on the nature of the theme being considered. Extracted data were stored in a Microsoft Excel file.

### Policy-maker consultations

We sought to identify senior health policy-makers (typically Directors and Deputy Directors, but including some Secretaries, Assistant Secretaries and Special Advisors) from a diverse group of LMICs, as well as a small number of international organization officials, to act as informants for this study. We started out by seeking to take advantage of two major global conferences, Health Systems Global 2016 (Vancouver, Canada), and the Prince Mahidol Awards Conference 2017 (Bangkok, Thailand), as venues where it would be possible to identify a diverse range of policy makers. The research team secured participant lists from both these meetings in advance of the meetings, and subsequently sent a total of 49 invitation letters to policy makers, both from the health sector and beyond. The study yielded 27 interviews across these two venues. Analysis of the distribution of respondents suggested that two regions in particular, Latin America and the Middle East, were not well covered and the study team sought collaborators (DM and FEJ) to enlarge the number of interviews in these regions, as well as supplementing the interviews already conducted with additional interviews in South Africa, India, and with international agency officials by phone and skype. In the Middle East, focus group discussions with small groups of policy makers were determined to be most efficient. We recognized that this method of sampling would not enable country-level saturation or cross-country comparisons, but believed that it would provide perspectives from a diverse group of key informants.

Before discussing the three themes, interviews began with an open-ended discussion of policymakers’ perceptions of health systems challenges in meeting the SDGs in their context, and policy changes being considered to mitigate these challenges. This was then followed by theme specific exploration that did not directly ask policy-makers for research priorities but rather asked about the kind of changes in policy and practice that they envisaged making in their country in response to the SDGs, and their related evidence needs. During analysis, we reframed policymaker comments on challenges and evidence needs as research questions.

With the permission of the respondent, interviews were recorded. Interviewers made extensive notes during and directly after the interview, referring to recordings where necessary. Responses in Arabic, French or Spanish were translated into English for analysis. A framework analysis approach [39] was employed: based upon notes and recordings, key findings concerning policy-maker views on the challenges to achieving the SDGs, likely policy changes required and potential evidence needs were extracted into a matrix for each of the three themes, as well as for the SDGs as a whole.

### Identification of research questions

All research questions and knowledge gaps from the overview of reviews were separated by theme and extracted into distinct Excel spreadsheets. Matrices from the policy-maker interviews were reviewed and where necessary, evidence needs were rephrased as research questions and inserted into theme-relevant spreadsheets. Through a systematic and iterative process of grouping and matching similar research questions, we transitioned from a large number of initial questions (ranging from 94 questions for Social Protection to 283 questions for Multisectoral Collaboration) to 30–36 distinct questions for each of the three themes. We targeted approximately 30 questions for each theme as, given the likely number of researchers participating in the online ranking exercise, this was thought to be an appropriate number to give reliable rankings.

The process of converting the problems and challenges identified by policy-makers into research questions was not an exact science, but relied considerably on the research team’s interpretation. Table 2 uses two examples to illustrate how policy-maker statements were combined with questions from reviews to result in over-arching research questions.

**Table 2:** Illustrative questions for the Social Protection theme showing how research questions from reviews and policy maker interviews were aligned

Final research question used in online priority setting process	Research questions identified in reviews	Policy-makers concerns
How can social protection programs be designed to minimize dependency and promote productivity amongst beneficiaries?	Need more longitudinal studies that examine duration of food aid and strategies for weaning off food aid. Studies with longer follow-up period were less likely to find significant effects of food aid [40].	SDG_IDI_SAFRICA “There’s a dependency issue. Need to graduate people from these grants to employment. We are trying to couple these grants with skills development.” SDG_FGD_Jordan “How do you incentivize people to become productive once they are on social protection and reduce dependency?” 1SDG_FGD_Jordan “Some continue to benefit from aid despite getting jobs.”
How effective are social protection programs in conflict affected settings in improving health outcomes and access to health services?	Need more high quality evaluations of the effects of social funds and community driven development initiatives in conflict-affected settings - including the impact of such programs on access to health services [41].	SDG_IDI_SOMALIA “What kind of mechanisms or schemes work in fragile states? How can these schemes provide protection for those who need it? What scheme is more relevant to Somalia now?” SDG_IDI_Liberia “Liberia is developing health for all scheme, with emphasis on vulnerable populations such as children, ebola survivors. There is a triple burden of war, ebola, other diseases. Social cash transfers would be very helpful. Major challenge is designing it and having a transparent way of delivering it, corruption is a big issue. Right now only have short term schemes.”

**Table 3:** Number of participating researchers and their contributions

	Social Protection	Multisectoral collaboration	Participatory and Accountable institutions
Number researchers invited	72	72	47
Number who participated	32	30	32
Dates of online consultation	4 September – 27 September 2017	2 October – 22 October 2017	14 August - 6 September 2017
Number distinct edits	207	79	81
Number of votes cast in second round	620	651	491

### Online priority setting process

Geographically and disciplinarily diverse researchers, with interests and prior experience in HPSR in LMICs, were identified through personal connections, the overview of literature reviews, and an open call for participants via email and Twitter. For each theme, we invited 50–70 health policy and systems researchers to participate, and 30–32 actually did so (Table 3). All participants were provided with a summary report on the overview of reviews, as well as with an Excel spreadsheet that showed how the proposed research questions had been synthesized from the reviews and policy-maker interviews. In their ranking, participants were asked to focus on the potential benefits or impact of research, but to also consider the tractability of the research question, and the extent to which answering it would benefit poor and marginalized communities.

Participation was online, using a platform called Codigital [42]. In the first round, participants refined the proposed research questions and voted on each other’s suggested edits. The study team then reviewed and incorporated proposed changes where appropriate, in some instances rejecting changes when it was felt that the fundamental nature of the question was being altered. In the second round, participants were presented with the revised questions in a series of pairwise, comparisons and asked to identify the higher priority question of the two. At the end of this process the final list of ranked questions was shared with participants in each of the three themes, and they were asked to provide feedback on the process and results.

### Webinars

We shared the results from all three themes through webinars hosted by the AHPSR and open to all those interested globally, including those who had participated in the online ranking exercise. The study team provided insight and background to the study method and results and invited policy makers to serve as discussants. The webinars were recorded and are available online on the AHPSR website. They served as a form of member checking, to understand how both the policy and research communities perceived the priorities identified, and as an opportunity to explore in more depth the priority research questions that emerged, and what it would take to address them.

## Results

### Policy-maker views on the SDGs and evidence needs in general

A total of 54 interviews (Table 4) and two focus group discussions (in which 10 policymakers from Bahrain and 17 from Jordan participated) were conducted. Most informants were from health sector government organizations, but some came from other institutions such as state governments, the office of the prime minister and departments of public service, the environment and planning. During the initial open-ended discussion about evidence needs for the SDGs, respondents identified current research needs in many of the domains that are already well-established within the HPSR field. For example, 15 policymakers, from diverse regions, spontaneously talked about the *health financing* challenges that they faced and evidence needs in this regard, 12 discussed *human resources for health* and 8 talked about the *private health care sector*. In terms of health financing, the primary concerns expressed were about how to expand health coverage and/or encourage greater enrollment in health insurance schemes. Several respondents, however, recognized ongoing low levels of government financing for health care, as well as the significant impact of financial shocks on health spending as being key challenges. On the issue of human resources for health, there were concerns about the numbers and uneven distribution of health workers, but a particular focus on poor skills within the health workforce. Seven policy-makers mentioned this, and several identified a lack of managerial skills as a particular problem.

**Table 4:** Profile of Policy-maker respondents in In-depth Interviews by region and language of interview

WHO Region	Total No. Respondents Identified & Invited	Total No. Respondents Included	Interview Language(s)
Africa Region	30	12	English
Region of the Americas	14	10	English, Spanish
South-East Region	18	14	English
European Region	0	0	NA
Eastern Mediterranean Region	5	4	English, Arabic, French
Western Pacific Region	15	8	English, Mandarin
Multi/Bi-lateral Org/NGOs	7	6	English

In addition to these three topics, which have been addressed by prior research priority setting processes [5, 6, 8], there was also substantive discussion of service delivery questions with 10 policy-makers spontaneously raising such issues. By far the strongest focus in this topic concerned the strengthening of primary health care, and in particular a shift from a more disease-oriented health system to one that focuses primarily on the prevention of disease. Several respondents noted that the growing burden of NCDs was accelerating this shift, and there was also interest in how best community initiatives and community outreach could be strengthened.

Of the three thematic areas addressed in this study, multisectoral collaboration was most frequently mentioned, with 32 policymakers mentioning this theme spontaneously and, of those, 15 policymakers identifying it as a priority. Broad concerns about the challenges of stimulating effective collaboration across sectors emerged; however, more specific concerns addressed whether or not other sectors adequately understood health sector needs and how their understanding could be enhanced. Other policymakers discussed the challenges of sharing data and other information across sectors. For example, one respondent observed that there is no single database which the government can use to analyse the array of different public services that households are using. Nine respondents talked about the links between equity, poverty, social protection and health, but the approach that they took and issues that they raised varied widely, for example, while two discussed the challenges to maintaining social solidarity and political support for targeting resources to the poorest, others were more concerned about potential abuse of social protection systems. Relatively few respondents (4) talked spontaneously about accountability and participation, and when they did, they tended to frame this as strengthening governance, and were particularly concerned with strengthening monitoring mechanisms and regular reporting from lower levels of the health system.

Finally, five respondents, all from low, or near low, income countries expressed concern that the MDGs had still not been met, and there was a danger that the SDGs would dilute the focus on this unfinished agenda.

### Ranked research questions within the focal themes

Table 5 shows the top ten research priorities that emerged from the online ranking exercise, reported under each of the three focal themes. In both the social protection theme, and the multisectoral collaboration theme there was a strong emphasis on practical implementation questions (ranked #1 and #8 for social protection and #1, #5 and #8 for multisectoral collaboration). While such implementation-focused questions were represented in the top ten for participatory and accountable institutions, they were ranked lower (at #8 and #10). Instead, the two top priority questions for participatory and accountable institutions focused on political factors affecting the adoption of accountability measures, as well as health worker reactions to such measures.

**Table 5:** Top 10 Research Questions across the three Focal Themes

Rank	Theme 1 – Social Protection	Final Score^a^	Theme 2 – Multisectoral Collaboration	Final score	Theme 3 – Participatory and accountable institutions	Final Score
1	How can social protection programs for health be designed, implemented, and evaluated to ensure sustainability and scalability in low and middle income countries, including conflict affected settings?	80.5%	Which strategies and mechanisms are effective in supporting the implementation of multi-sectoral collaborations for health? (e.g., enabling legislation, policy mandate, decentralized control, accountability and incentive mechanisms, dedicated resources, training/skill development, etc.)	68%	What political factors (e.g. the discretionary power of health providers, politicization of the health system and other political factors) mediate the adoption or effectiveness of accountability initiatives (e.g. digital technology, health committees, local media or more informal citizen actions)?	69%
2	What are the contextual factors that influence the effectiveness of conditional and unconditional cash transfer schemes for health?	71.1%	What factors are necessary for sustaining multi-sectoral collaborations over time?	63%	What processes and incentives (e.g. financial/non-financial, punitive/trust-building, learning loops, peer review) facilitate the acceptability of accountability mechanisms among frontline healthcare providers?	68%
3	How can various social protection initiatives be best integrated or harmonized across sectors?	66.7%	How does the use of evidence differ across different sectors and how can we make health evidence more accessible and actionable in other sectors?	63%	What reforms (e.g. decentralized budgeting, performance based financing) in the governance of national health systems are most likely to enhance provider accountability to consumers and in what contexts?	68%
4	How cost-effective are CCT programs compared to supply-side interventions (e.g. strengthening quality of infrastructure and expanding services) in improving health?	65.9%	What is the role of community-based partnerships and initiatives in driving multi-sectoral collaborations for health?	63%	What mechanisms and contextual/historical factors enable or hinder various actors (MoH officials, lay and professional health workers themselves, clients & communities, civil society, private sector, religious groups providing healthcare) to interact productively in order to improve accountability and responsiveness?	63%
5	What are the impacts of social protection programs in conflict affected settings and their effectiveness in improving health outcomes and access to health services?	63.6%	What types of leadership, partnership, and governance structures and processes are most effective for multisectoral collaboration	60%	What conditions or factors are necessary to enable accountability initiatives to address issues at the macro (e.g. political social, cultural and economic environment), meso (e.g. organizational culture, incentives), and micro (e.g. individual ethics, rationalizations) levels?	58%
6	What are the pathways through which social protection programs affect clinical and nonclinical outcomes, and what are the implications on program design?	61.8%	What are the key challenges to implementing multi-sectoral programs and interventions to address health issues (e.g., food security, NCDs, HIV/AIDS)?	60%	What are the impacts (expected and unexpected) of accountability interventions on the health workforce? (E.g. attitudes, behavior, practices, morale, decision-space, service provision, corruption, performance etc.)	58%
7	What is the impact of social protection initiatives on health equity outcomes and equitable access to quality health care services for poor and marginalized populations?	61.4%	How do contextual factors such as institutional arrangements, governance arrangements, democratic values, partnership experiences affect the success (or failure) of multi-sectoral collaborations?	53%	What is the impact (expected and unexpected) or effectiveness of transparency and accountability interventions on various aspects of governance and health system performance (e.g. healthcare quality, service utilization, human resource management, corruption, participatory decision-making, and citizen-state relationships within and beyond the health sector)?	58%
8	How can routine information systems be strengthened and used to monitor and evaluate social protection systems for health?	57.5%	How can we best improve the capacity of stakeholders involved in multi-sectoral action for health (such as health advocates, or health practitioners), to engage in and also promote multi-sectoral initiatives?	53%	How can citizen monitoring and evaluation be effectively integrated into health system planning and implementation?	58%
9	How do social protection programs influence the interaction between public and private health care providers with regards to service availability, quality of care and utilization?	55.6%	Which study designs and methods are best suited to understanding multisectoral collaborations, their governance, functioning, and outcomes?	52%	How do specific contexts (e.g. political environment, strength of democracy, social cohesion/heterogeneity, level of economic inequity, health system privatization) influence the potential for success/failure of particular types of accountability initiatives?	58%
10	What are the effects of unconditional cash transfer programs on healthcare quality, coverage and outcomes across settings in low and middle income countries?	55.0%	How do multi-sectoral collaborations affect health equity and social determinants of health?	51%	What tools and design features (e.g. format, frequency of use, degree of standardization) can enhance the effectiveness of accountability initiatives, such as digital reporting tools, report cards, social audit tools/social autopsy tools, community report on outbreak responses etc.?	58%
10			How do interventions that target non-health Sustainable Development Goals affect health outcomes?	51%		

^a^The final score reflects the percentage of times which this question, when paired with another question, was preferred by the participant

Understanding how context affects different types of health systems interventions was important across all three themes, ranking #2 for social protection, #4 and #9 for participatory and accountable institutions, and #7 for multisectoral collaboration. Respondents reflecting on social protection concerns, seemed particularly interested in how social protection systems might help protect health and promote access to health services in conflict-affected settings.

Questions regarding the effectiveness of different interventions were included in the top ten questions across all three themes (questions #5 and #7 for social protection, #9 and #10 for multisectoral collaboration and #6 and #7 for participatory and accountable institutions), but overall, they were ranked lower than more operationally-oriented questions.

### Webinars

The webinars lasted between 1 and 1.25 hours and attracted between 75 and 150 registrants each. Participants included experienced researchers, decision makers, students as well as funders.

Policymakers attending the first webinar on *Health system social accountability research priorities for the SDG era* (https://hsgovcollab.org/en/news/webinar-iii-what-research-needed-advance-accountability-health) raised the concern that although NGO-led initiatives have been shown, in some cases, to support accountability in communities, they tend to lack sustainability. There was therefore an emphasis on the importance of sustaining and institutionalizing accountability within health systems. Policymaker participants urged researchers to think beyond frontline health workers, and to instead consider the accountability of stakeholders and structures at higher levels of the system.

Discussants also noted that ‘accountability’ should be conceptualized as a long term and multifaceted process rather than a short term, bounded intervention; and that although technology may play a role in supporting accountability in the health system (through online complaints mechanisms, for example) it should not be assumed that technology can and will automatically empower citizens; and if it does, there still remain questions around equity of access. Hence, those working in this area were urged to pay attention to risks associated with accountability initiatives (e.g., individuals facing backlash), incentives for change, and stakeholder perspectives on accountability. This is due, oftentimes to a deep distrust between various stakeholders (patients and providers, different levels of the health bureaucracy). Therefore, identifying realistic incentives and supportive contexts that enable improved accountability will be vital.

During the webinar on *Multisectoral collaboration for health: what are the priorities? (*https://www.who.int/alliance-hpsr/events/msc-priorities-webinar/en/), discussants noted that efforts in multisectoral collaboration not only involve stakeholders across sectors (such as health, education etc.) but also across institutional types (e.g., public, private, CSOs, etc.), as well as administrative levels (e.g., national, state/provincial, local). These various dimensions, and their cultural and contextual differences, therefore should be explicitly considered when planning, implementing, assessing, and writing about multisectoral collaborations. Issues such as conflicting interests (for example between ministries of health and tobacco or alcohol industries) introduce additional challenges that impact different sectors differently. Establishing a shared vision and trust among stakeholders was consistently highlighted as a prerequisite for effective multisectoral collaboration which suggests that substantial investment of time and effort and, in some cases, a new paradigm of thinking (e.g., moving away from “command-and-control” bureaucratic processes) may be required.

Participants also noted that a common language, terminology, and frameworks would help develop a shared understanding within and across sectors and disciplines, for policymakers, practitioners, and researchers. Such a foundation may facilitate more robust evidence about multisectoral collaboration and henceforth, conversations advancing these deliberations should include non-health actors.

Panelists on the *Social protection for health: What are the health policy and systems research priorities?* webinar (https://www.who.int/alliance-hpsr/events/social-protection-priorities-webinar/en/) discussed the fact that many low and middle income countries are making increased investments in social protection, which are often intended to have positive health effects, yet the methodological basis for determining priorities for investment in health vis a vis other sectors is not well established. While the speakers recognized that much of the extant work on social protection addressed conditional cash transfers, they suggested that there were new challenges which such transfers could be applied to (such as obesity) and that further research to understand how conditional cash transfer programs could support the ‘nudges’ proposed by behavioral economics was warranted. Overall the webinar reinforced concerns about the lack of dialogue between those working on HPSR and researchers with a primary focus on social protection and poverty alleviation.

## Discussion

### HPSR priorities

Paying heed to the interconnectedness between the various systems impacting health as highlighted by the SDGs, this study aimed to identify HPSR priorities for the SDGs in relation to three key themes: (i) Protecting and promoting access to health services through systems of social protection (ii) Strengthening multisectoral collaborations for health and (iii) Developing more participatory and accountable institutions [34–36].

However, as we look across the entire study, we note that the policy maker consultations revealed other policy challenges and evidence needs associated with the SDGs, beyond the three themes we focused on. Of the three themes that we selected as foci for this study, two were perceived by policy-makers to be of relatively high importance, with a particular emphasis on multisectoral collaboration, and a lesser emphasis on social protection. While the theme of participatory and accountable institutions was less commonly discussed in detail by policymaker respondents, it is possible that civil society representatives and research funders would have expressed greater interest in this theme. Outside of the three themes studied here, policymakers had a continuing interest in health financing topics, human resources for health and the private sector. Questions about how to reorient health systems towards public health and disease prevention also emerged as a priority.

The work of the previous Task Force on Health Systems Research to achieve the MDGs provided recommendations in more general terms than this paper, seeking not to identify specific research questions, but rather illuminate a number of important issues [4]. Indeed, there are close links between two of our themes, and the priority areas identified in the Task Force report, namely approaches to intersectoral engagement for health, and governance and accountability. Though likely relevant to the MDGs, the reorientation of health services towards a more preventative and public health perspective was not identified as a priority by the Task Force, perhaps because the MDGs did not focus on NCDs. Further, while the Task Force report highlighted health insurance, and equitable health care, it did not consider broader linkages with systems of social protection.

Within the focus of participatory and accountable Institutions the most highly prioritized questions were policy analysis questions that sought to understand how politics and power influenced the scope for successfully implementing strategies to create more accountable health sector institutions [34]. Furthermore, the importance of context, process and implementation factors that mediate or influence accountability initiatives stood out. While policy and political analyses have been relatively neglected within HPSR [43], for a topic such as accountability, it is clear that understanding the complex and political dimensions of change is critical.

With respect to the theme of multisectoral collaborations, policymakers’ evidence needs centered on fundamental questions about how to make MSCs work, addressing practical questions such as how to structure, govern, lead, implement and sustain MSCs, as well as how these factors vary across different types of collaborations and the barriers and facilitators to effective MSCs. By contrast, the most commonly mentioned need from the overview of reviews (representing a researcher viewpoint) was identifying appropriate study designs and methods for understanding MSCs. Both of these sets of issues - the very practical questions as well as questions concerning research methods - emerged as priorities in the priority setting process [35] Taken together, these research priorities are indicative of a field of study in fairly early stages of development; they also point to the need for greater clarity on the types of research methods best suited to answering the pragmatic research questions that are most important to policymakers.

The theme of social protection brought scale and sustainability of social protection to the fore as priorities [36]. Furthermore, vulnerable populations (disabled, children, refugees, migrant workers, the elderly, and those suffering from domestic violence) as well as distributional effects were of particular concern amongst policymakers. Policymakers also described concerns around how to identify beneficiaries in the context of limited data systems, and how to subsequently prevent abuse of systems of social protection and encourage beneficiaries to graduate from such programs. With most research questions around social protection focusing on the practicality of implementation and its potential effects, there appears to be an enhanced need for collaboration between HPSR researchers and those working directly on social protection systems.

While it is beyond the scope of this paper to discuss in detail the type of research necessary to address the research priorities that emerged from this exercise, there are a number of points worth highlighting. First, across all three themes, the specific priority questions identified through this process emphasize the need for implementation research. That is, research that occurs within real world contexts and seeks to help policymakers and practitioners understand factors (including context) that influence implementation, as well as informing implementation strategies [44]. Second, for two of the themes (Social Accountability and MSC) it was apparent that lack of conceptual clarity was a barrier to quality HPSR. The domain of social accountability does not lack relevant theories of frameworks - quite the opposite - but the research team noted the need to build a “shared conceptualization” around different notions of accountability that would facilitate comparison of results across different settings [34]. By contrast, MSC has very few suitable frameworks or theories associated with it, and stronger theoretical development is necessary to support work in this field. Finally, the findings of this study underscore the need for multidisciplinary collaboration in HPSR. In social accountability, much of the seminal work has been done by experts in public administration or political science. In social protection, there is a very clear need to bridge the gap between health systems researchers and the economists, political scientists and labour experts working in this space.

### Priority setting methodology strengths and limitations

We adapted Ranson and Bennett’s [9] HPSR priority setting methodology to include an online process for researchers to refine and rank questions. In an era where accountability and transparency are the bedrock for trusting relationships between researchers and decision-makers, a priority setting exercise that involved decision-makers and researchers from around the world was important. In previous priority setting exercises this step of the process has been carried out in a face-to-face setting, which probably allows more meaningful dialogue to refine the research questions, and more thoughtful rankings. Replacing the face-to-face meeting with an online exercise, however, allowed us to reduce costs, and encourage broader participation. Although the research questions were derived from academic and policymaker sources, we only invited academic experts to participate in the ranking, based on our assumption that these stakeholders would best be able to rank research questions according to priority and feasibility. However, future efforts may benefit from continuing to involve policymakers not only in generating research questions but also in ranking them. While it seems unlikely to us that policymakers would participate in an online ranking exercise, sharing our findings with them via the webinars was illuminating, and we believe that closer engagement with them during the final stages of such a research priority setting exercise is key.

The work described here has several limitations. First, while this exercise aspired to be relevant across LMICs, in reality the number of policymakers interviewed means that there were many perspectives and ideas likely not captured in this exercise. It is notable that while one of our themes focused on multisectoral collaboration, this exercise involved relatively few policymakers outside the health sector and exploring the views of such stakeholders may have offered other viewpoints. Further, policymakers are typically more comfortable expressing challenges that they face rather than research needs, and so the process of reframing challenges into research questions entailed some subjective judgement by the research team. Ideally, additional rounds of this process would have involved further member checking with policymaker informants to understand whether the priorities that emerged resonate with their thinking.

## Conclusions

In many respects the complex and wide-ranging SDGs represent a paradigm shift from the straightforward and focused MDGs. We believe that to-date, the research community, including those working on HPSR, have not fully processed the implications of the SDGs. Our work found that many of the existing priority areas for HPSR continue to be of great importance to policymakers seeking to achieve the SDGs: there continues to be a pressing need for country-level analyses of health financing mechanisms, strategies for strengthening the health workforce, as well as approaches to strengthening primary health care arrangements. Given the volume of research conducted in these themes over the past 10 years, it is likely also necessary to build stronger linkages between researchers and policymakers so as to ensure that ongoing research addresses policy-relevant questions and reaches the ears of policymakers.

Beyond the well-established HPSR priority areas, the SDGs emphasize the inter-connectedness and complexity of the world in which we operate. The broad and multifaceted nature of the SDGs aligns well with the ethos of HPSR, but also suggests new foci for research. For example, the growing understanding of the interconnectedness between human health, animal health and environmental health, and the systems created to track and respond to challenges in these sectors, has stimulated increasing interest in multisectoral collaborations for health. To-date much of this work is narrowly focused on specific topics or health issues, but HPSR need to address questions around the institutions, systems, capacities and governance structures that can support sustainable, multisectoral, collaboration. Similarly, systems of social protection are intimately entwined with health, from offering financial protection to those struck by ill health (through disability payments, or welfare schemes), to enabling the health sector to identify and target services at those most in need. Until now, much of the HPSR conducted within this theme has addressed conditional cash transfer schemes, but our analysis identifies a much broader array of relevant topics for HPSR. Finally, while the Task Force on Health Systems Research [4] highlighted the importance of governance and accountability within health systems, this theme did not feature strongly within the MDGs. By contrast, within the SDGs, Goal 16 emphasizes the need for effective, accountable and inclusive institutions. It appears that there is much that still needs to be understood in order to ensure effective investments in institutional strengthening.

The research priorities identified here may be informative to multiple stakeholders:

- Funders: these results provide a strong foundation for future investments by global as well as regional funders of health research.
- National and sub-national governments: we encourage governments and other country level stakeholders to review these research priorities to determine which are important within their respective contexts. This step would help drive research alignment with national policy and evidence needs, promoting the relevance of research conducted.
- HPSR community: the research agenda reflected here has significant implications both in terms of the nature of research as well as how this research is conducted. While studies of effectiveness continue to be important, the prioritized research agenda emphasizes implementation research that will need to engage communities, health workers and other stakeholders in its execution.
- Intersectoral actors: the nature of all three prioritized themes highlights a need to draw non-traditional stakeholders into HPSR, whether this is decision-makers from other sectors, those working in systems of social protection, or representatives of civil society.

The SDGs should provide a further impetus for change in how we plan, execute and communicate HPSR.

## Data Availability

The qualitative datasets generated and/or analysed during the current study are not publicly available due difficulties in anonymizing the data collected, and concerns about protecting the anonymity of respondents, but are available from the corresponding author on reasonable request.

## Abbreviations

AHPSR: Alliance for Health Policy and Systems Research
HPSR: Health Policy and Systems Research
LMIC: Low or middle income country
MDG: Millennium Development Goals
MSC: Multisectoral collaboration
SDG: Sustainable Development Goals
UHC: Universal Health Coverage
WHO: World Health Organization
